# Effects of workday characteristics and job demands on recovery from work among Finnish home care nurses: a multi-source cross-sectional study

**DOI:** 10.1007/s00420-023-02026-y

**Published:** 2023-11-30

**Authors:** Visa Väisänen, Salla Ruotsalainen, Pihla Säynäjäkangas, Satu Mänttäri, Jaana Laitinen, Timo Sinervo

**Affiliations:** 1 Finnish Institute for Health and Welfare, Helsinki, Finland; 2 Finnish Institute of Occupational Health, Oulu, Finland

**Keywords:** Recovery, Nurses, Job demands, Home care, Care needs

## Abstract

**Objective:**

Ageing populations and poor care workforce availability are causing increasing job demands for home care nurses across Europe. While recovery from work helps sustain work ability and wellbeing, past research has relied mainly on self-reported measures of health, stressors, and recovery. This study aims to examine how objective and subjective job demands are associated with measured day-time recovery among home care nurses.

**Methods:**

Heart rate variability recording was conducted for 95 Finnish home care nurses. The study participants documented their work tasks throughout the workday and filled a wellbeing questionnaire. The amount of care time, breaktime, number of different weekly clients, and their care needs were obtained from the survey. The associations between job demands and measured day-time recovery were analysed using multivariate linear regression.

**Results:**

The amount of day-time recovery was on average 75 min. The number of different clients during the workday (e.g., care continuity) and higher care needs of the clients were associated with lower day-time recovery. Additionally, something slightly disrupting the course of the workday was associated with increased recovery.

**Conclusions:**

Our findings indicate that reducing especially the objective job demands (workday characteristics) can contribute to better day-time recovery among home care nurses. To help sustain work ability and improve wellbeing, day-time recovery can be promoted with better work scheduling that supports care continuity and ensures sufficient care resources and support for nurses with many clients or clients with high care needs.

## Background

Population ageing is a European wide phenomenon, and many countries will face a substantial increase in the number of older people in the future (Eurostat 2023). Furthermore, alongside other European countries, Finland is currently suffering from a lack of care personnel working in the health and social services, and due to the increasing number of older people, the workforce situation will deteriorate even more in the coming years. As such, Finland is a fitting country to study how to meet care challenges that arise from aging populations. Recent studies conducted in home care have demonstrated problems among caregivers, with time pressure and stress as well as dissatisfaction and turnover intentions being prevalent (Gebhard & Wimmer 2023; Ruotsalainen et al. 2020). Furthermore, demands for high efficiency, and insufficient number of nurses can result in increasingly intense workdays, which might lead to strain, poor recovery, and sickness absences. Insufficient recovery causes load cumulation and, consequently, may lead to health issues over time (Sluiter 1999; Wentz et al. 2020).

Care for older people has shifted towards home-based care in Finland during the last decades (Ministry of Social Affairs and Health 2020). Finnish care policy has diminished the role of institutionalised care, rather focusing on non-institutionalised care, such as assisted living facilities and home care. Home care in Finland comprises of home nursing and home help services (Social Welfare Act 2014). Currently, home care mainly focuses on helping with activities of daily living and medication. Home care is mostly given by practical nurses with three years of vocational training, while registered nurses focus more on clinical care and administrative duties. Consequently, approximately 80 percent of the care workforce in home care are practical nurses and only 13 percent registered nurses (Väisänen et al. 2022). However, house-keeping tasks such as cleaning are not included in the work of home care nurses, rather those services are contracted. Due to policies stressing home care, the number of individuals receiving home care and the number of visits have greatly increased during the last decades, whereas the number of nurses in home care has not followed (Kehusmaa & Alastalo 2022).

The lack of personnel, in addition to lower average functioning and growing number of clients in home care have caused an increase in job demands by increasing the number of client visits, but also through more intense and less flexible workdays (Kaihlanen et al. 2023; Ruotsalainen et al. 2020). The increase in the number of clients and visits has led to several nurses visiting one client (Väisänen et al. 2022), which might compromise the continuity of care. Better continuity of care may also improve workers’ wellbeing, since previous research has associated increased continuity of care with positive outcomes, such as reduced turnover intentions (Tourangeau et al. 2017). Furthermore, previous studies have demonstrated that high job demands may have detrimental effects on health (Häusser et al. 2010), as well as on recovery (Geurts & Sonnentag 2006) of the employees. Job demands, for example role conflicts, might lead to turnover of personnel (Nei et al. 2015), which in case of a labor shortage is undesirable. Lastly, daily care work especially in home care may face sudden changes, for instance via absence of a colleague or the need for longer visits in case of a client’s worsening condition, which can in turn drastically influence the course of a workday. Such events may lead to increased stress and lower wellbeing among workers (Bono et al. 2013). Little attention has been paid to the care needs of clients and their worsening condition as a stress factor. More dependent clients are cared for at home (Väisänen et al. 2022), which may increase the stress and worry of employees.

In order to reduce the negative outcomes of job demands, a day-to-day recovery process, mainly occurring during leisure time is needed. Recovery from occupational stress is an important part in replenishing resources and restoring work ability after strain and fatigue (Geurts & Sonnentag 2006; J K Sluiter et al. 2003; Zijlstra & Sonnentag 2006). More importantly, sufficient recovery from work prevents imbalanced autonomic regulation and, therefore, elevated cardiovascular disease risk (Thayer & Lane 2007). Although most of the recovery occurs during sleep (Geurts & Sonnentag 2006), day-time recovery, also during the workday, plays an important role in improving outcomes in self-rated health and work engagement (de Bloom et al. 2015), in addition to enabling after-work energy balance readjustment and recovery (Demerouti et al. 2012). Workers often employ different strategies that enable day-time recovery during the workday, such as physical and mental microbreaks, and work-related energy management (de Bloom et al. 2015). All of these strategies, however, might not be possible in home care duties, where a predetermined work schedule is often optimised in order to conduct client visits as efficiently as possible.

Recently, recovery during sleep, and factors affecting it, have been quite extensively studied (e.g., Zijlstra and Sonnentag 2006; Togo and Takahashi 2009). However, there are limited numbers of studies on the determinants of recovery occurring during worktime, especially from the health and social care sector. Furthermore, while the negative impact of high job demands on care workers’ wellbeing are widely documented, it seems that the composition of workday (e.g., the proportion of direct care time or the number of clients) and clients’ care needs and their association on recovery are yet scarcely explored. Earlier studies have focused mainly on self-reported stress factors and outcomes, for instance time pressure and stress. There is very little research taking into account clients’ care needs and clients’ functioning, especially in home care.

In this study, we aim to examine which factors affect day-time recovery (as defined via 24-h heart rate variability measurements) with a study design combining work time measures from a time-allocation study, clients’ care needs, and subjective measures related to job demands.

The following research question was formulated:

How are different objective (workday characteristics) and subjective job demands (perceived stressors) associated with measured day-time recovery among home care nurses?

## Methods

The study design was cross-sectional, using multiple sources of data. In total, 95 home care nurses from nine different Finnish public home care units volunteered to participate in the study. The units ranged in size from 18 to 44 in the number of nurses and on average 11 nurses participated from each unit. The care units for the time measurement study were recruited from the Finnish RAI benchmarking network and the volunteering care units for the physiological measurements were located in two large cities (six units) and in three smaller municipalities (three units). The municipalities were situated in two regions in southern Finland. Data were collected during one week in October 2021.

Physiological measurements were taken using Firstbeat Bodyguard-2 (Firstbeat Technologies Ltd, Finland) attached before the work shift (for a more detailed description of the measurements, see Mänttäri et al. 2023). The participants were either in the morning shift (*n* = 81) or in the evening shift (*n* = 15). The morning shifts started approximately at 7:00 and ended after 14:00, while the evening shifts started around 15:00 and ended at 22:00. The measurement included a 24-h heart rate and heart rate variability recording. In addition, during the workday, the study participants continuously filled a time measurement survey, which included each task’s start and end times, in addition to the client’s name. Furthermore, a brief wellbeing questionnaire was included. Data were merged with client information from the Finnish Resident Assessment Instrument (RAI) register. To obtain information about the clients’ care needs, the Case Mix Index (CMI) of the Finnish RUG-III/18-classification in RAI was used.

As the sample size was relatively small, pairwise deletion was used to handle missingness. The number of missing values for the six variables of interest varied from 11 to 0, being on average 5.7. Most missing data were in the wellbeing questionnaire on questions relating to the disruptions during the workday (*n* = 11) and role conflicts (*n* = 10). In the end, 17 registered nurses and 79 practical nurses were included in the study, of whom all were female.

### Variables

#### Measured day-time recovery

Measured day-time recovery was determined through the heart rate variability measurements. Day-time recovery refers to a physiological state where the participant is not under physical or mental stress during the time spent awake. The state of recovery is determined based on heart rate and heart rate variability, and recovery is determined to be occurring when heart rate is reduced and heart rate variability is increased. (Firstbeat Technologies 2014). The amount of day-time recovery was measured in minutes throughout the whole day, including both the workday and spare time, but not during sleep.

In addition, to describe the physical demands of care work, mean metabolic equivalent (MET) values from the duration of the workday were used in the descriptive analyses. METs are commonly used to express the intensity of physical strain as a multiple of the resting metabolism, with a value of one corresponding to the metabolism of a resting person (Ainsworth et al. 2011). Day-time recovery and MET were analysed with Firstbeat Lifestyle Assessment software (Firstbeat Technologies Ltd, Finland).

Maximal oxygen consumption in ml/kg/min (VO_2_ max), which is a commonly used indicator of cardiovascular fitness, was used as an adjusting variable in the analyses. Body-weight relative VO_2_max was calculated based on self-reported background variables (age, sex, height, weight, and habitual activity level) with a non-exercise-based formula (Jackson et al. 1990).

#### Objective job demands

Workday characteristics (objective job demands) were determined from the self-administered time measurement survey. Workday characteristics measured included the amount of direct care time, the amount of breaktime, number of different clients, and the mean CMI value of clients.

Direct care time was measured as a proportion of care time throughout the workday, where the person was in contact with a client. The proportion of direct care time was calculated from the length of the workday, which varied from person to person.

Breaktime was measured as a proportion of the workday, where the worker had documented being on a break or lunch. The proportion of breaktime was calculated from the length of the workday, which varied from person to person.

Number of different clients refers to the number of unique clients throughout the week and was used as an indicator on the level of continuity of care. As nurses had different number of workdays, the number of different clients through the work week was divided by the number of workdays, which was multiplied by five to get the average number of different clients per week.

The mean CMI of clients was calculated using the day’s clients and their Case Mix Index values of the Finnish RUG-III/18-classification (Väisänen et al. 2022). CMI value directly corresponds to the care needs of the client, measured in terms of cost adjusted care time required. To illustrate, a nurse’s clients that had an average CMI of 1.15 require approximately 15 percent more care time than a nurse’s clients with an average CMI of 1.0.

#### Subjective job demands

Subjective job demands were determined from the wellbeing survey, answered at the beginning and at the end of the work shift. In addition to other topics, the survey included questions on work-related demands and wellbeing. Subjective job demands measured included perceived time pressure, role conflicts, and whether something disrupted the workday.

Time pressure was a sum variable of two items (Spearman-Brown coefficient: 0.86): *“I did not have enough time for clients”* and *“I did not have enough time to perform work properly”* (Harris 1989).

Role conflicts variable was formed of two variables (Spearman-Brown coefficient: 0.57): *“I receive an assignment without adequate resources and materials to execute it”* and *“I have to buck a rule or policy in order to carry out an assignment”* (Rizzo et al. 1970).

Both time pressure and role conflicts were asked on whether the mentioned thing disturbed, worried, or strained you today, rated on a five-point scale (1 = never, 2 = a little, 3 = somewhat, 4 = often, 5 = very often).

Whether something disrupted the workday was determined via a single item question on assessing today’s workday with three possible answers: *“Workday went as planned”*, *“Workday went nearly as planned”*, and *“Something disrupted the course of the workday”*. Due to the question layout, the item was treated as a categorical variable with “workday went as planned” as the reference group.

### Statistical analyses

Sample characteristics were described using percentages, means, and standard deviations. The relationships between measured day-time recovery and various job demands were analysed using bivariate and multivariate ordinary least squares (OLS) linear regression analysis.

First, bivariate linear regression analysis was performed separately for each of the independent variables with measured day-time recovery as the dependent variable.

Next, the multivariate models with measured day-time recovery as the dependent variable were built in three steps, first entering objective job demands (direct care time, break time, number of different weekly clients, and mean CMI of the clients). In the second model, only subjective job demands (time pressure, role conflicts, workday disruptions) were included. Lastly, both objective and subjective job demands were entered in the model. The multivariate models were adjusted for age, VO_2_max, and occupational status (registered or practical nurse).

The multivariate models were validated and assessed using the following metrics: R-squared, F-statistic, and AIC/BIC values (Akaike Information Criterion/Bayesian Information Criterion). No multicollinearity was detected between the variables, as the Variance inflation factor (VIF) values were at maximum 2.5. Normality of the data, checked with Q–Q plots, was adequate. In addition, according to a residuals vs. leverage plot, no significant influential outliers were found.

Statistical significance was defined as *p* < 0.05. Statistical analyses were performed using R Statistical Software version 4.2.2 for Windows 10 (R Core Team 2020).

### Ethical considerations

A written consent was obtained from the participants who agreed to participate in the study. The participation was voluntary, and the participants were informed of the possible discomforts (mainly skin irritation caused by applying the electrodes) of the study. Further, it was highlighted that if they wished to withdraw from the study, they could do it in any point of time. The ethical approval was obtained from the Ethics Committee of the Finnish Institute for Health and Welfare (THL/1447/6.02.01/2021).

## Results

### Sample characteristics

Characteristics of the study participants can be seen in Table 1. In our sample, the proportion of practical nurses was 82% (*n* = 62), whereas the proportion of registered nurses was 18% (*n* = 14). All participants were female, with an average age of 42. The nurses had been in their current work position on average for 9 years. Workdays were on average 7 h and 45 min in length (including breaks).

**Table 1:** Sample characteristics of the study participants (*n* = 96)

Variable	All (*n* = 96)
	Mean (SD)
Age	42.2 (12.1)
Female	100%
Practical nurse	72%
Registered nurse	18%
Years in current work position	8.7 (8.7)
Physiological measurements	
Day-time recovery (minutes)	75.3 (82.3)
Metabolic equivalent (MET)	1.80 (0.52)
Maximal oxygen uptake (VO_2_max)	28.5 (8.0)
Workday characteristics	
Direct care time, % of total workday	41.3 (19.8)
Direct care time (minutes)	192.6 (97.1)
Break time (minutes)	28.3 (21.0)
Number of different weekly clients	19.3 (8.4)
Mean CMI^1^ of clients	1.08 (0.26)
Wellbeing survey	
Time pressure (1–5)^2^	2.0 (1.0)
Role conflicts (1–5)^2^	1.4 (0.7)
Workday went as planned	35%
Workday went nearly as planned	47%
Something disrupted the workday	18%

^1^Case Mix Index. Corresponds to the care needs of the client, measured in terms of cost adjusted care time required

^2^Assessed on a scale from 1 ‘never’ to 5 ‘very often’

The amount of day-time recovery varied between 0 and 380 min, being on average 75 min. In general, the physical workload of the participants, measured with metabolic equivalent (MET), was rather low (1.8, SD 0.5) indicating light intensity (Ainsworth et al. 2011). For a more in-depth descriptive analysis of the physiological measurements of Finnish home care nurses, see study by Mänttäri and co-authors (Mänttäri et al. 2023).

The amount of direct care time was on average 41 percent, which translated to 3 h and 13 min. The number of different clients during the week was 19. The average care needs of the clients were slightly higher than the average client in home care (mean CMI: 1.08). According to the wellbeing survey, time pressure was reported as moderately low (2.0, SD: 1.0) and role conflicts were reported as low (1.4, SD: 0.7). One third of the nurses’ workdays went as planned, with a majority reporting them going nearly as planned (47%) or something disrupting the workday (18%).

### Regression analysis

Results of the bivariate and multivariate linear regression analysis can be seen in Table 2. In the bivariate models, higher objective job demands indicated lower measured day-time recovery. All objective job demands, except breaktime, were significantly associated with day-time recovery, with the number of different weekly clients having the strongest effect. Of the subjective job demands, higher time pressure was strongly associated with lower measured day-time recovery, while role conflicts and disruptions during the workday were not. Age, occupational status, or VO_2_max were not associated with day-time recovery.

**Table 2:** Bivariate and multivariate linear regression analysis of associations of objective and subjective job demands on measured day-time recovery in minutes

Variable	Bivariate models^1^		Model 1 objective multivariate		Model 2 subjective multivariate		Model 3 final multivariate	
	Stand beta	*p* value	Stand beta	*p* value	Stand beta	*p* value	Stand beta	*p* value
Intercept			0.13	0.316	− 0.13	0.527	− 0.11	0.630
Direct care time	− 0.19	0.065	− 0.27	0.099			− 0.29	0.091
Break time	0.13	0.214	− 0.02	0.894			− 0.02	0.862
Number of different weekly clients	− **0.22**	**0.028**	− **0.31**	**0.018**			− **0.36**	**0.026**
Mean case mix index of clients	− **0.22**	**0.037**	− **0.26**	**0.019**			− **0.25**	**0.048**
Time pressure	− **0.28**	**0.010**			− **0.35**	**0.021**	− 0.19	0.221
Role conflicts	− 0.18	0.110			0.03	0.817	0.08	0.639
Day went as planned (ref.)								
Day went nearly as planned	0.08	0.744			0.44	0.125	**0.64**	**0.033**
Something disrupted the day	− 0.58	0.069			− 0.25	0.549	0.07	0.877
Age	− 0.06	0.548	− 0.07	0.620	− **0.32**	**0.046**	− 0.31	0.066
Maximal oxygen uptake (VO_2_max)	− 0.04	0.966	− 0.03	0.816	− 0.26	0.102	− 0.15	0.379
Registered nurse (ref. practical nurse)	0.16	0.540	− 0.47	0.211	0.12	0.699	− 0.59	0.154
Adjusted *R*^2^			*R*^2^ = 9.1%		*R*^2^ = 11.5%		*R*^2^ = 19.2%	
F-statistic (*p* value)			2.27 (*p* = 0.037)		2.47 (*p* = 0.025)		2.62 (*p* = 0.008)	
AIC/BIC			259/282		233/255		220/251	

^1^Bivariate models were calculated for each variable (dependent variable: measured day-time recovery). The results are presented row-wise, with one model per variable

Statistically significant results (*p* < 0.05) marked in bold

In the first multivariate model with objective job demands, higher number of different clients and their higher care needs (mean CMI) were associated with lower day-time recovery among nurses. In the second multivariate model with subjective job demands, only time pressure was significantly associated with day-time recovery.

In the last multivariate model with all variables, the number of different clients and their care needs were statistically significantly associated with measured day-time recovery. While the amount of direct care time was not statistically significantly associated with day-time recovery, the results indicate a possible weak association. Of the subjective job demands, only disruptions were partly statistically significant, with the response “day went nearly as planned” being associated with higher day-time recovery. The background variables of VO_2_max and occupational status were not associated with day-time recovery in any of the multivariate models. However, the results indicate that older nurses could have lower measured day-time recovery.

## Discussion

In this study, we examined, using multiple sources of data, how different objective and subjective job demands were associated with day-time recovery among home care staff. The results indicate that lower number of different clients and their lower care needs (CMI) are associated with higher amount of day-time recovery. In addition, nurse’s workday going nearly as planned (as opposed to going as planned) was associated with higher amount of recovery. Other subjective job demands were not associated with day-time recovery. Based on the findings of our study, it further seemed that care work in home care is not particularly physically demanding, which was also determined in a study by Mänttäri and co-authors (Mänttäri et al. 2023).

Firstly, it is important to note that the overall level of day-time recovery had significant variance (0–380 min). According to the 24-h HRV measurements, some participants had no day-time recovery, while others had multiple hours. Different job descriptions, daily task lists, and quality of spare time might explain some of the variance. A workday with densely scheduled client visits, limited break time, followed by a busy evening could potentially lead to a valid measurement with 0 min of recovery. On the other hand, a registered nurse working mostly in the office, having relatively few client visits and small number of stressful events, followed by a quiet evening, could feasibly have over 6 h of day-time recovery. However, while recovery-focused physiological measurements using the Firstbeat Bodyguard2-devices have been determined as reliable and valid (Palmer et al. 2021; Parak & Korhonen 2013), it is possible that, for example, accuracy of the recovery analysis and inter-individual differences might have affected the results.

Our findings suggest that home care nurses with more direct care time with clients, which may indicate more intense work, might have less day-time recovery, since the results indicated a weak association. The results further suggested that clients’ care needs affect the recovery of nurses, as nurses with clients that required more resources had lower amount of day-time recovery. This might be partly explained by the fact that clients with higher care needs require additional care time and more complex care tasks, which might for instance hasten subsequent client visits, leaving only little or no time for a break. These results are in accordance with the findings from previous studies, which have highlighted the association between job demands and poor recovery (Gifkins et al. 2020; Sonnentag et al. 2022). Due to the lack of care workforce, more efforts should be placed on strategies to arrange nurses’ time for recovery. In addition, methods and skills that promote day-time recovery from work should be utilised and developed. While the population ages throughout Europe, the demand for nurses will further increase. Consequently, it is crucial to at minimum maintain the work ability of nurses working in older people care. Deteriorating working conditions can lead to high turnover of nurses, resulting in lower care availability and quality (Antwi & Bowblis 2018), which would be a major public health concern.

The finding regarding the association between clients’ higher care needs and nurses’ lower recovery is especially relevant, as to our knowledge no studies have been able to account for the case-mix in this setting. However, similar results have been found in other nursing populations, for instance regarding patient-related stressful situations in emergency care (De Wijn & Van Der Doef 2020). The care needs of the clients should be considered when planning daily visits and coordinating care, because it might be associated with client safety. Sufficient care resources should be ensured for clients with high care needs, requiring for instance assistance from more than one nurse. The objective demands may reflect the findings related to subjective demands as well. An association between time pressure and lower recovery was found, however, the association did not remain significant after including the objective demands into the model. This might be explained by the correlation between direct care time and time pressure, which are both indicators of workday intensity. Still, the subjective stress cannot be ignored, as the decisions for example relating to turnover, are made based on subjective experiences. A former study showed that objective demands (staffing level) was a strong instrument for job demand and strain (Elovainio et al. 2015). Although the lack of staff is a major cause of job demands, the perceived demands also seem to have their effect on strain, or in this case, on day-time recovery.

Regarding care continuity and day-time recovery, we found that nurses with higher number of different clients per week had lower amount of recovery. On one hand, this can simply relate to the intensity of the workday, as the number of different clients was correlated with the number of total clients through the day. On the other hand, the discovered lower recovery might relate to a lack of job control, where a nurse is unable to plan their day and might have to visit more unfamiliar clients. The results regarding job control are in line with previous research on nurses and other occupations, which indicates that increasing the level of job control might promote better recovery from work (Gifkins et al. 2020; Kinnunen & Feldt 2013). If the number of different clients per week is low, the clients are more familiar to the nurses, which means that the continuity of care is high. This enables understanding the specific needs and wants of each client, their treatments, and where to find different things at their homes. In terms of recovery, higher continuity of care might refer to more ‘routine-like’ work, which may enhance recovery during workday via familiarity of environments and clients. In addition, better continuity of care in home care has been associated with better patient outcomes, which can help save care resources and improve quality of care (D. Russell et al. 2011).

One unexpected finding was that the amount of breaktime did not contribute to higher recovery in our study. Previously, the positive association between within-day work breaks and recovery has been found both among nurses and among the general population (Sagherian et al. 2023; Sianoja et al. 2016). Nurses in our study had on average only 30 min of breaks during their workday, which could be considered relatively low. It is possible that in home care work securing sufficient and uninterrupted breaks during the day can be difficult, which might have affected the results. If the breaks were often fragmented or disturbed by for example phone calls or consultations, their effect on recovery can diminish. Securing adequate break time during workday is important, since studies have shown that fragmented or too short breaks are associated with burnout (K. Russell 2016), and break disturbances with leaving intentions (Wendsche et al. 2022). Lastly, as the level of work autonomy has been shown to moderate recovery occurring during lunch breaks (Trougakos et al. 2014), it is possible that breaktime not being associated with day-time recovery can be partly attributed to low job control of home care nurses.

Another surprising finding was that smaller disruptions during the workday can potentially increase the amount of measured day-time recovery. It is possible that some disruptions might be relatively small in their effect and may instead lead to additional break-time, for instance in form of waiting for clients or other nurses. The potential of within-working day recovery in preventing stress and maintaining performance is in the observation that recovery is not equal to rest (Kinnunen et al. 2011). Therefore, by including familiar routine-like work tasks to the workday, it is possible to influence employees’ day-time recovery. As demonstrated in a study from Bono and co-authors (Bono et al. 2013), positive events during workday were associated with lower stress. In our study, we were not able to distinguish whether the disruptions were positive or negative. The negative aspects of brief disruptions, such as a busier schedule for rest of the day, seemed to not have a significant effect on day-time recovery, yet they might contribute negatively to perceived job demands and work-related wellbeing.

Age and physical fitness (VO_2_max) were used as adjusting variables in the multivariate models. Individual factors, such as age, sex, physical fitness, body composition, and health status have been found to affect heart rate variability (Shaffer & Ginsberg 2017; Teisala et al. 2014). However, in this study, physical fitness did not have an effect on measured day-time recovery, whereas the results indicated a tendency for lower day-time recovery in older nurses. This might be due to the finding that work in home care is not especially physically straining, and therefore psychosocial risk factors instead might be more prevalent. In comparison, work in hospitals and assisted living facilities might involve more physical demands, such as moving patients (Poole Wilson et al. 2015). Mänttäri and co-authors (Mänttäri et al. 2023) found that physical workload in relation to maximal capacity was higher for older nurses in Finnish home care, which was attributed to age-related decline in physical fitness. Higher relative physical workload could also explain the lower day-time recovery in older nurses in the current study. The VO_2_max values used as an indicator of physical fitness were calculated based on self-reported background variables and are, therefore, only estimates, which might also explain the lack of association. Nevertheless, literature indicates that maintaining adequate physical fitness is important for reducing relative occupational workload and ensuring sufficient recovery from occupational stress (De Bloom et al. 2018; Sliter et al. 2014).

### Strengths and limitations

Our study had a few distinct advantages. First, the multi-source study design with objective and subjective job demands as explanatory variables, in addition to physiological outcome data, offered a novel way of exploring the effects of different job demands on recovery among home care staff. Second, heart rate and heart rate variability monitoring using ambulatory method and performed during authentic work time provides reliable and valid results on physical workload (Smolander et al. 2008, 2011), and recovery (Palmer et al. 2021; Parak & Korhonen 2013). The objective measurement further diminishes the biases related to self-estimation.

Next, the inclusion of the workday characteristics, obtained from a comprehensive time measurement survey and clients’ RAI data, offered insight into the direct and objective job demands home care nurses experience. Additionally, we were able to take into account the clients’ need for care, which is seldom done. This is not a minor aspect, as in this study the mean of clients’ case mix varied substantially, indicating that clients of some nurses may require significantly more care time than others.

Last, as opposed to the number of participants often found in physiological measurement studies, in our study an adequate number of participants was recruited. Regardless, the statistical power of the analyses was slightly limited, which caused some of the probable associations not to be statistically significant. In order to control for missingness in our data and analyses, we used pairwise deletion to make the most out of the dataset.

However, our study had some limitations. First, while the workday characteristics consisted of objective measurements of job demands, the data were collected using relatively complex self-reported surveys. According to Lopetegui and co-authors (Lopetegui et al. 2014), the use of self-reporting in time motion studies can result in overestimating care time and in discrepancies on how nurses fill the surveys. These factors were mitigated with video tutorials and training sessions organised for the participating care units. In addition, the nurses reported the start and end times for each task, which may diminish the risk of overestimating care time. This same time code in regard to care time is used also in the official nurse documentation systems.

Some factors exist which can affect the validity and the reliability of the results. First, there exists a possibility for selection bias, as the care units were selected on a volunteer basis. This means that units with no excess time for research activities, and possibly worse workforce or client situation, might not be included in the study. As such, the units included in the study might be in a better situation in terms of care workforce and overall job strain. Next, in terms of statistical validity, some of the independent variables (especially the survey questions), were positively skewed. Sensitivity analysis was conducted with differently categorized variables: The direction of the results remained, but the effect of something slightly disrupting the day often weakened to statistical non-significance.

It is unclear how the results will transfer to other care settings, as the work in home care is considerably more mobile and independent compared to for example work in nursing homes. In addition, countries with significantly differently organised care for older people might have other factors that affect care workers job demands and recovery from it. Last, due to the cross-sectional study design, causality cannot be inferred from the statistical analyses.

## Conclusions

Our findings suggest that especially objective job demands, measured through workday characteristics, appear to be the main drivers in hindering day-time recovery throughout the day. Interventions aimed at ensuring day-time recovery throughout the workday should focus mainly on the characteristics of the workday. For example, work scheduling should be conducted in a way that can enable better continuity of care and allocate sufficient time for nurses with many clients or clients with higher care needs. This way, the recovery of nurses during work and spare time can be supported, which can help maintain work ability and ultimately improve work-related wellbeing. Continuity of care can provide periods of recovery via routine-like work with familiar clients and environments. Furthermore, it seems that nurses might utilise minor disruptions in the work as sort of microbreaks, which can contribute to day-time recovery and complement formal breaktime. Ageing populations require adequate care workforce, which must be maintained by improving the wellbeing of nurses and supporting the day-time recovery from work.

## Data Availability

The datasets generated during and/or analysed during the current study are available from the corresponding author on reasonable request.

## Abbreviations

CMI: Case mix index
MET: Mean metabolic equivalent
RAI: Resident assessment instrument
SD: Standard deviation
VIF: Variance inflation factor
VO_2_max: Maximal oxygen consumption
